# Alzheimer Disease Associated Loci: *APOE* Single Nucleotide Polymorphisms in Marmara Region

**DOI:** 10.3390/biomedicines12050968

**Published:** 2024-04-27

**Authors:** Aya Badeea Ismail, Mehmet Sait Dundar, Cemre Ornek Erguzeloglu, Mahmut Cerkez Ergoren, Adem Alemdar, Sebnem Ozemri Sag, Sehime Gulsun Temel

**Affiliations:** 1 Department of Medical Genetics, Faculty of Medicine, Near East University, 99138 Nicosia, Cyprus; aya.badeea@gmail.com (A.B.I.); mahmutcerkez.ergoren@neu.edu.tr (M.C.E.); 2 Department of Electrıcal and Computer Engineering, Graduate School of Engineering and Sciences, Abdullah Gul University, 38000 Kayseri, Türkiye; msaitdundar@erciyes.edu.tr; 3 Halil Bayraktar Health Services Vocational School, Erciyes University, 38030 Kayseri, Türkiye; 4 Department of Translational Medicine, Institute of Health Sciences, Bursa Uludag University, 16059 Bursa, Türkiye; cemreornek@gmail.com (C.O.E.); ademalemdar@uludag.edu.tr (A.A.); 5 Department of Medical Genetics, Faculty of Medicine, Bursa Uludag University, 16059 Bursa, Türkiye; ozemri@uludag.edu.tr; 6 Department of Histology & Embryology, Faculty of Medicine, Bursa Uludag University, 16059 Bursa, Türkiye

**Keywords:** *APOE* gene, Alzheimer’s disease, variant frequency, Turkish population

## Abstract

Alzheimer’s disease (AD) is a major global health challenge, especially among individuals aged 65 or older. According to population health studies, Turkey has the highest AD prevalence in the Middle East and Europe. To accurately determine the frequencies of common and rare *APOE* single nucleotide polymorphisms (SNPs) in the Turkish population residing in the Marmara Region, we conducted a retrospective study analyzing *APOE* variants in 588 individuals referred to the Bursa Uludag University Genetic Diseases Evaluation Center. Molecular genotyping, clinical exome sequencing, bioinformatics analysis, and statistical evaluation were employed to identify *APOE* polymorphisms and assess their distribution. The study revealed the frequencies of *APOE* alleles as follows: ε4 at 9.94%, ε2 at 9.18%, and ε3 at 80.68%. The gender-based analysis in our study uncovered a tendency for females to exhibit a higher prevalence of mutant genotypes across various SNPs. The most prevalent haplotype observed was ε3/ε3, while rare *APOE SNPs* were also identified. These findings align with global observations, underscoring the significance of genetic diversity and gender-specific characteristics in comprehending health disparities and formulating preventive strategies.

## 1. Introduction

Alzheimer’s disease (AD) is a neurodegenerative disorder correlated with progressive dementia in the senior population. Worldwide, at least 55 million people are believed to be living with Alzheimer’s disease or other dementias [1]. The diagnostic characterization of AD disease is the allocation of extracellular amyloid beta (Aβ) forming amyloid plaques and the intracellular compiling of hyper-phosphorylated Tau protein eliciting neurofibrillary tangle formation, leading to neural and glial cell loss [2]. Alzheimer’s disease (AD) is divided into early-onset (EOAD) and late-onset (LOAD). EOAD, occurring before age 65, is linked to mutations in *APP*, *PSEN1*, and *PSEN2* genes, constituting <1% of familial cases. LOAD, accounting for 95% of cases, is associated with the apolipoprotein E gene (*APOE*) as a major risk factor [3,4,5].

The human *APOE* on chromosome 19q13.2 encodes apolipoprotein E, which plays a key role in regulating cholesterol and lipid metabolism. It facilitates lipid distribution to neurons in the central nervous system by binding to APOE cell surface receptors LDLR (the low-density lipoprotein receptor) and LRP1 (LDL Receptor Related Protein 1) [6]. The *APOE* has three allelic variants: ε2 (Cys112, Cys158), ε3 (Cys112, Arg158), and ε4 (Arg112, Arg158), resulting from single nucleotide polymorphism (SNP) substitutions. These variants have negative implications on protein function [7]. The *APOE* ε4 allele is a major genetic risk factor, increasing AD risk up to 15-fold when homozygous, and adversely affecting lipid profiles and cardiovascular health. Conversely, the rare ε2 allele is often considered protective, while ε3 is generally regarded as neutral for AD, lipid profiles, and cardiovascular health [8,9].

Individuals carrying a single copy of APOE ε4 have a threefold higher risk of developing the disease compared to those with both copies of ε3 [10]. Postmortem brain biopsy analysis in the elderly revealed a stronger correlation between upregulated amyloid beta disposition and the presence of APOE ε4 than ε2 [11]. Research conducted on mice with abnormal *APOE* gene has indicated that the absence of *APOE* expression leads to disruptions in lipoprotein profiles. These disruptions contribute to the development of cardiovascular disease and neurological disorders, ultimately resulting in a shorter lifespan for the mice [12].

The *APOE* gene is considered a genetic marker for osteoporosis, as it plays a role in transporting liposoluble molecules like vitamins K and D to bone osteoblasts. The APOE ε4 allele is positively linked to low bone mineral density (BMD), accelerating bone loss and increasing fracture risk. Conversely, the APOE ε2 allele is associated with significantly lower bone loss, and the ε3 allele is considered protective [13,14].

Epidemiological studies in the Mediterranean countries demonstrated Turkey leading the highest numbers of AD in the Middle East and Europe [15].

In this study, our primary objective was to investigate the frequencies of common *APOE* gene SNPs (single nucleotide polymorphisms) associated with Alzheimer’s disease, specifically rs7412 and rs429358. Additionally, we aimed to examine the frequencies of nine less common *APOE* SNPs (c.11G > A rs373985746, c.42C > G rs440446, c.137T > C rs769452, c.651C > T rs72654468, c.555C > T rs781722239, c.55G > A rs563571689, c.447G > C rs762933906, c.434G > A rs267606664, c.920C > T rs770562611).

The selection of these specific SNPs was based on their established associations with Alzheimer’s disease and their relevance to the Turkish population residing in the Marmara region. Other SNPs may also be relevant to the disease, but were not included in the scope of this particular study. The data can contribute to our understanding of the genetic landscape related to Alzheimer’s disease in this specific population and potentially provide insights into disease risk and susceptibility.

## 2. Materials and Methods

### 2.1. Study Design and Participants

The genetic data we used in the current retrospective study was retrieved from the total of 588 random patients consisting of 360 female and 228 males that were consulted in our Medical Genetic Diseases Diagnosis Center. All participants were Turkish citizens residing in the South Marmara region, precisely Bursa city. All procedures performed in this study were in accordance with the ethical standards of the institutional research committee and with the 1964 Helsinki declaration and its later amendments or comparable ethical standards (approval number: YDU, 2020/80-1120). An informed consent form was taken from all participants.

### 2.2. Molecular Genotyping from Clinical Exome Sequencing

The genetic data obtained as a result of the clinical exome sequencing analysis performed for the patients who came to Bursa Genetic Evaluation Center for diagnostic purposes were analyzed anonymously and retrospectively. From these cases, genomic DNA was extracted from 3.8 mL of peripheral venous blood collected into Na-EDTA vacutainers (BD Vacutainer; BD Diagnostic Systems, Istanbul, Turkey) using the QIAamp^®^ DNA Mini Kit (QIAGEN, Ankara, Turkey) for routine diagnostics. The Clinical Exome Solution (CES) was performed using exome enrichment, whereas all procedures were carried out according to the manufacturer’s protocols (SOPHiA GENETICS, Lausanne, Switzerland). In total, 4900 genes with known inherited disease-causing mutations were captured based on a target enrichment paired-end sequencing kit that was performed on an Illumina NextSeq 500 system with a read length of 150 × 2. Base calling and image analysis were conducted using Illumina’s Real-Time Analysis software (v2.19). The BCL (base calls) binary is converted to FASTQ utilizing the Illumina package bcl2fastq.

### 2.3. Bioinformatics Analysis and Statistical Analyses

The clinical exome sequencing data was reanalyzed to detect the presence of *APOE* polymorphisms. All bioinformatics analysis were performed on the Sophia DDM^TM^ platform which includes algorithms for alignment, calling SNPs and small Indels (Pepper), calling copy number variations (Muskat) and functional annotation (Moka). Raw reads were aligned to the human reference genome (GRCh37/hg19). Variant filtering and interpretation were performed on Sophia DDM^TM^. Integrative Genomics Viewer (IGV) [16] was used to facilitate file visualization. The allelic frequency of the *APOE* polymorphisms was calculated by dividing the count of the allele of interest by the total number of alleles at that specific genetic locus in the studied population. This calculation provided an estimate of the relative prevalence of the allele. For statistical analysis involving demographic variables, Microsoft Excel spreadsheet software v2403 (Microsoft Corporation, San Jose, CA, USA) was utilized. The sample sizes for females and males were determined by summing the number of individuals in each group. Additionally, summary statistics such as the mean, mode, and median were calculated for age, providing an overview of the age distribution in the sample.

## 3. Results

### 3.1. Demographics of Study Participants

The study population consisted of a total of 588 randomly selected patients who sought consultation at the Bursa Medical Genetic Diseases Diagnosis Center. The participants included 360 females and 228 males. None of the patients had been diagnosed with neuropathological disorders, such as Alzheimer’s or dementia. All participants were Turkish citizens residing in the South Marmara region, specifically in Bursa city, which is located on the northwest coast of Turkey. The age and gender of the study participants are shown in (Table 1).

### 3.2. APOE Alleles Frequencies

The study identified the presence of two well-known *APOE* single nucleotide polymorphisms (SNPs) associated with Alzheimer’s disease: rs429358 (ε4 allele) and rs7412 (ε2 allele). The frequencies of these alleles were 9.94% and 9.18%, respectively, while the wild-type ε3 allele was estimated to have a frequency of 80.86%. The study also examined another *APOE* gene polymorphism, rs440446, which is located in the intron 1 enhancer region. The frequency of this polymorphism was estimated at 70.068%. The overall allele frequencies of the mentioned polymorphisms in patients from Bursa Province, Turkey, are shown in (Table 2).

### 3.3. APOE Genotype Distribution: Gender-Specific and Overall Analysis

The data presented in Table 3 provides a comprehensive breakdown of genotype distribution within the general population, categorized by gender and focusing on specific genetic variations (rs7412, rs440446, rs429358). Notably, females consistently exhibited a higher prevalence of mutant genotypes across all three SNPs compared to males. In rs7412, females had a higher percentage of heterozygote mutants (CT), while males had higher percentages of homozygote wild types (CC). For rs440446, there was a significant discrepancy in the distribution of homozygote wild types (CC) between males and females, with females presenting a higher percentage. In rs429358, female carriers exhibited a higher prevalence of heterozygote mutants (TC) and homozygote mutants (CC) compared to males.

Further analysis involved exploring various combinations of these genetic variations within individuals’ profiles. For rs7412/rs440446 (CT/CG), the total occurrence was 7.7% in males, while females had a notably higher prevalence at 48.8%. Similarly, the combined genotype for rs7412/rs429358 (CT/TC) had a total occurrence of 1.7%, with a prominent prevalence in females at 70.0%. The absence of certain combined genotypes, such as CT/TT and TT/TT, indicates their rarity in the studied population. Among the individuals examined, 105 people carried one copy of the ε4 allele, while only 6 individuals had two copies of this allele. In contrast, 102 individuals were heterozygote for the ε2 allele, and only 3 individuals were homozygote. For the third genetic variation, c.42C > G rs440446, approximately 340 individuals had one copy, while around 242 individuals had two identical copies of this variation.

These findings highlight the gender differences in genotype distribution and prevalence of specific genetic variations within the general population, as well as the occurrence of different combinations of these variations.

### 3.4. APOE Haplotype Distribution

In addition to analyzing genotype distribution, we also conducted an analysis of *APOE* gene haplotype (E2/E2, E2/E3, E2/E4, E3/E3, E3/E4, and E4/E4) based on the rs429358/rs7412 genetic variations, with a focus on categorizing them by gender. This analysis provides information on the percentages and average ages associated with each haplotype within the specified genetic variations as seen in (Table 4). The genetic data reveals that the most prevalent genotype in the general population is E3/E3 (TT/CC), accounting for 65.97% of the sample, with an average age of 26.45 years. E2/E2 (TT/TT) and E2/E3 (TC/TC) are represented in 0.51% and 15.64% of the population, respectively, with average ages of 33 and 25.97 years. E2/E4 (TC/TC) accounts for 1.71% of the population, with an average age of 15.9 years. E3/E4 (TC/CC) and E4/E4 (CC/CC) are found in 16.16% and 1.02% of the population, respectively, with average ages of 28.97 years for E3/E4 and 30.67 years for E4/E4. When examining the table by gender, females exhibit a higher prevalence of the E3/E4 (TC/CC) and E4/E4 (CC/CC) genotypes compared to males. The average age is relatively consistent across different genotypes, ranging from 30.53 to 50 years in females. Males, on the other hand, show a higher prevalence of the E2/E2 (TT/TT) genotype compared to females. E2/E3 (TC/CC) accounts for 19.74% of males, while E3/E3 (TT/CC) is the most prevalent genotype at 64.9%. The average age varies across different genotypes in males.

Additionally, the study identified additional non-common APOE SNPs in the study group, although their frequencies were very low. SNPs such as rs373985746, rs563571689, rs72654468, and rs769452 had estimated allele frequencies of 0.17%. Similarly, rs781722239, rs762933906, rs267606664, and rs770562611 were estimated at 0.085%.

## 4. Discussion

Determining human genetic variations among different populations worldwide helps scientists understand the link between diseases and ancestry groups making the process of developing effective treatment easier [17]. A few studies have been previously conducted investigating *APOE* gene polymorphism in the Turkish population. For example, Malle and colleagues calculated the *APOE* and *APOA-IV* allele frequencies, respectively, in 240 non-related Turkish subjects living in Germany with central and eastern Anatolia origin [18]. According to their findings, the *APOA-IV* allele frequency in the Turkish participants living in Germany is similar to that of a Hungarian population. Furthermore, they discovered that the prevalence of allele ε2 and allele ε4 is among the lowest reported worldwide, with frequencies of 0.048 and 0.067, respectively. While the *APOE* ε3 allele frequency was reportedly the highest, with the frequency of 0.885, at the expense of both ε2 (0.084) and ε4 (0.074) which exceeds known high frequencies in a Japanese population [19]. So, while their overall allele frequencies most closely resemble those disclosed for the Japanese, the hypothesis in their study was additionally supported by the low ε2 (0.054) and ε4 (0.070) allele frequencies in a Greek-Cypriot population of Cyprus with an approximate ethnic composition of 80% Greek Cypriots, 18% Turkish Cypriots and 2% foreigners [18,20]. Another study in the adult Turkish community by Komurcu-Bayrak [21] and his colleagues focused on the two functional promoter SNPs in the *APOE*, 219G/T (rs405509) and +113G/C (rs440446), involved in transcriptional activation of the *APOE* reported that the frequencies of the ɛ2/ɛ3, ɛ2/ɛ4, ɛ3/ɛ3, ɛ3/ɛ4, and ɛ4/ɛ4 genotypes were 9.8%, 1.2%, 75.9%, 12.5%, and 0.6%, respectively, in their study population. While the frequencies of alleles ε2, ε3, and ε4 in the adult Turkish community were 0.055, 0.871, and 0.074, respectively [21].

The current study revealed the presence of the two most prevalent APOE SNPs associated with AD namely (rs429358 and rs7412) at a frequency of 0.0994 and 0.0918, respectively. Along with another *APOE* SNP known as the non-classical intron 1 enhancer of the *APOE* (rs440446) at a frequency of 0.70068. The comparative allele frequencies of three major SNPs across diverse populations and ethnic groups are listed in Table 5.

In our study, the frequency of ε2 (0.0918) and ε4 (0.099) alleles was greater than the allele frequency reported in the Turkish population by Malle and colleagues (1996) [18] and Komurcu-Bayrak and colleagues (2011) [21] but ε3 results were quite similar. It is also greater than frequencies reported in Greek Cypriotes [20]. However, the ε2 allele frequency in our study is remarkably comparable to prior ε2 frequencies identified in the Chinese and Norwegian populations (0.097 and 0.90, respectively) [19]. Africa and Oceania have higher levels of ε2 allele frequency 0.099 ± 0.083 and 0.111 ± 0.052, respectively. Similarly, ε4 allele averages were greater in Oceania 0.221 ± 0.149 and Africa 0.209 ± 0.090, while ε3 allele frequencies were highest in Indian and Asian groups. ε2 allele showed a statistically significant decrease in North Asian populations, while ε4 showed a substantial increase in North European populations [23].

Our analysis involved a comparison between our genetic data on SNPs (rs440446, rs7412, rs429358) and the frequency results documented in the recently established Turkish Genome Project Data Sharing portal (TÜSEB). Remarkably, the outcomes closely mirrored our own findings, with respective frequencies of 0.601, 0.068, and 0.068 (https://tgd.tuseb.gov.tr/en/) accessed on 12 February 2024. The gender-based genotype analysis of (rs7412, rs440446, and rs429358) in our study group revealed significant patterns of females consistently exhibiting higher percentages of mutant genotypes across all three SNPs. In rs7412, females were more heterozygote mutant (CT) carriers, while males were homozygote wild types (CC) carriers. In rs440446, the distribution of homozygote wild types (CC) was predominant particularly in females. As for rs429358, female carriers demonstrate a higher prevalence of both heterozygote mutants (TC) and homozygote mutants (CC) compared to males. Additionally, the combinations of these genetic variants within individuals’ profiles were also analyzed. The combined frequency of rs7412/rs440446 (CT/CG) was 7.7% in males and 48.8% in females, while rs7412/rs429358 (CT/TC) were at 1.7%, in males and 70.0% in females. The limited number of specific combination genotypes, such as CT/TT and TT/TT, was shown by their absence in the study group. These findings emphasize gender-specific importance in genotype distributions and potential implications for genetic associations with these specific SNPs. The observed gender differences in genotype distribution found in (Table 3) may be influenced by hormonal influences, genetic predispositions, X-chromosome inactivation in females, sample size and population bias. Hormones, such as estrogen and testosterone, can affect gene expression patterns, while genetic factors and X-chromosome inactivation may contribute to gender-specific variations. Additionally, sample composition and potential bias, as well as interactions with environmental factors, could further influence genotype frequencies.

*APOE* haplotype (ε2/ε2, ε2/ε3, ε2/ε4, ε3/ε3, ε3/ε4, ε4/ε4) distribution was also examined based on rs429358/rs7214 and categorizing by gender. ε3/ε3 (TT/CC) was the most common in the general population. Females had higher prevalence of E3/E4 and ε4/ε4, while males had higher prevalence of ε2/ε2 and ε2/ε3. Apart from the three major SNPs we also detected other rare *APOE* SNPs. Despite their extremely low frequencies, we were able to find additional non-common *APOE* SNPs in our research population. The frequencies of the alleles for rs373985746, rs563571689, rs72654468, and rs769452 were calculated to be 0.17%. Furthermore, the frequencies of rs781722239, rs762933906, rs267606664, and rs770562611 were calculated to be 0.085%. The comparative allele frequencies of non-common *APOE* SNPs across diverse populations and ethnic groups are listed in Table 6.

Most of the non-common SNPs show very minor variation across different populations; rs781722239, rs762933906, rs267606664, rs770562611 are all most frequent in the European population. While rs781722239 and rs770562611 have rare to zero frequency. Our results for rs781722239 and rs267606664 are similar to other populations. While our rs373985746 results are similar to those from east-Asia. Unlike other populations where rs781722239 and rs770562611 are rare, our study includes these SNPs, presenting a distinctive feature in our dataset. Looking into the characteristics associated with each of the non-common SNPs, we found that rs373985746 is involved in the AD modulation process [24]. Rs72654468 and rs781722239 were both found to be associated with CVD abnormalities [24,25]. Rs769452 and rs267606664 were found to be involved in neurodegenerative and CVD abnormalities as well as free cholesterol and low-density lipoprotein measurements. It is also is reported as a marker for familial-type AD and muscular atrophy, respectively [26,27]. Lastly, rs762933906 and rs770562611 have no record of being pathogenic. We conducted a comprehensive review to analyze APOE data within the healthy population of Turkey and compare the findings with our own results. From the total sample size of 1008, six articles and a thesis yielded findings closely aligned with our own data as seen in Table 7 [28,29,30,31,32,33].

During the course of the study, it was discovered that many of the same individuals had two or three polymorphisms at the same time. The varying allele frequencies might be due to the population’s distinct genetic background and the limited sample size. Additionally, the genetic traits of the Marmara region in Turkey may deviate from global norms. Beyond *APOE*, Alzheimer’s disease is associated with MTHFR and ACE polymorphisms. Folate insufficiency connects with MTHFR and the 677 T allele frequency. Adequate folate intake is found to be correlated with the prevalence of the 677 T allele, seen in Europeans and the Americas. Malnutrition and viral disorders impacting folate absorption lower the 677 T gene prevalence in African Zambians [34,35]. Similarly, Diet and lifestyle can also alter *APOE* gene variant prevalence in different ethnic groups [36]. Therefore, continuous studies on *APOE* SNPs rates in other populations is necessary. The pathogenic role of *APOE* gene ε4 in Alzheimer’s disease (AD) is still debated, with some studies suggesting a toxic gain of function in its interaction with Aβ, while other effects may result from the loss of protective function [37]. Interestingly, blocking the Aβ/APOE interaction may enhance Aβ clearance and reduce plaque deposition, challenging the hypothesis that it would lead to increased plaque formation [38]. The former statement is supported by the emergence of Aducanumab, a treatment targeting amyloid aggregates, offering a promising avenue in Alzheimer’s disease therapy [39]. *APOE* gene variants are associated with Alzheimer’s disease through mechanisms involving amyloid β metabolism, tau pathology, lipid metabolism, neuroinflammation, vascular factors, and gene-environment interactions. Additional factors like genetic diversity, population-specific genetic factors, and health disparities further shape the context of Alzheimer’s disease in Turkey.

## 5. Conclusions

In conclusion, in our study, *APOE* gene variants (rs42935, rs7412, and rs440446) align with African, Asian, European, and European Finnish populations. ε2 and ε4 alleles were less common than ε3. The genotype and haplotype analysis of our study revealed a tendency for females to exhibit higher percentages of mutant genotypes as well as higher prevalence of ε3/ε3. Our study identified rare *APOE* SNPs as well. Genetic variation aids in understanding health disparities and crafting effective prevention. However, distinct Turkish genetic traits are notably underrepresented in public databases which hinders accurate interpretation of genetic variations, diagnosis of genetic disorders, and research on Turkish-specific genetic factors. APOE’s interaction with amyloid β (Aβ) is crucial in Alzheimer’s disease pathogenesis. Potential pharmacological interventions based on our data include developing drugs to modulate *APOE* expression/function and targeting Aβ aggregation or clearance pathways. This study has several limitations that need to be acknowledged. Firstly, the sample size was limited, which might have hindered the identification of additional *APOE* SNPs. Secondly, there was a dissimilarity in age groups between males and females, which calls for caution, as age-related variations could potentially influence the results. Therefore, it is important to conduct future studies with larger sample sizes and ensure similar age groups or more diverse range of ages among the targeted populations. Complementing AD-associated genetic variant studies with follow-up data and clinical correlations is crucial for a comprehensive understanding of their implications in the disease. Additionally, although the study was conducted in Marmara, the most populated region in Turkey, to obtain a more comprehensive understanding of genetic diversity and regional differences in Turkey, future studies should encompass multiple regions.

## Figures and Tables

**Table 1:** Age and gender of study participants.

Total General Population	Female	Male
n = 588	n = 360	n = 228
**Age distribution (years)**		
Mean = 27	Mean = 31	Mean = 19
Median = 20	Median = 34	Median = 15
Mode = 4	Mode = 4	Mode = 3

**Table 2:** The frequencies of *APOE* alleles within the Turkish population residing in the Marmara region.

*APOE* Allele	Transcript Name	rs Number	c.DNA	No. of Allele	Total No. of Allele	Allele%
*APOE* ε2	NM_000041	rs7412	c.526C > T	108	1176	9.18%
*APOE* ε3	NM_000041	rs429358/rs7214	c.388T/T	951	1176	80.86%
*APOE* ε4	NM_000041	rs429358	c.388T > C	117	1176	9.94%

**Table 3:** Gender-specific genotype distribution: exploring distinct genetic variations in the general population.

Allele and Genotype Distribution			General Population			Female			Male		
		Genotype	(n)	%	Age (avg)	(n)	%	Age (avg)	(n)	%	Age (avg)
	General Population		588	100	27	360	61.2	31	228	38.8	19
rs440446	wt homozygote	CC	6	1.0	22	4	66.7	25	2	33.3	15
rs440446	mut heterozygote	CG	340	57.8	27	206	60.6	32	134	39.4	19
rs440446	Mut homozygote	GG	242	41.2	27	150	62.0	31	92	38.0	19.3
rs429358	wt homozygote	TT	477	81.1	26	282	59.1	31	195	40.9	19,4
rs429358	mut heterozygote	TC	105	17.9	28	75	71.4	32	30	28.6	17
rs429358	Mut homozygote	CC	6	1.0	31	3	50.0	31	3	50.0	30
rs7412	wt homozygote	CC	483	82.1	27	305	63.1	31	178	36.9	20
rs7412	Mut heterozygote	CT	102	17.3	25	54	52.9	32	48	47.1	17
rs7412	Mut homozygote	TT	3	0.51	33	1	33.3	50	2	66.7	25
rs7412/rs440446	mut hetero/mut hetero	CT/CG	45	7.7	23	22	48.8	31	23	51.1	15
	mut hetero/Mut homo	CT/GG	57	9.7	27	32	56.1	33	25	43.8	19
	Mut homo/Mut homo	TT/GG	3	0.5	33	1	33.3	50	2	66.6	25
	TOTAL		105	17.9	24	55	52.4	33	50	46.7	17
rs7412/rs429358	mut hetero/mut hetero	CT/TC	10	1.7	16	7	70.0	19	3	30	8
	mut hetero/Mut homo	CT/TT	0	0.0							
	Mut homo/Mut homo	TT/TT	0	0.0							
	TOTAL		10	1.7	16	7	70.0	19	3	30	8
rs7412/rs429358	mut hetero/mut hetero	CG/TC	46	7.8	29	30	62.2	35	16	34.7	17
	mut hetero/Mut homo	CG/CC	0	0.0							
	Mut homo/Mut homo	GG/CC	6	1.0	31	3	50	31	3	50	30
	Mut homo/mut hetero	GG/TC	55	9.4	28	43	78.2	31	12	21.8	18
	TOTAL		107	18.2	28	76	71	32	31	29	19
rs7412/rs440446/rs429358	TOTAL	CT/GG/TC	10	1.7	16	7	70	19	3	30	8

**Table 4:** *APOE* haplotype distribution by gender and rs429358/rs7214 genetic variations.

Genotype		E2/E2	E2/E3	E2/E4	E3/E3	E3/E4	E4/E4
(rs429358/rs7214)		TT/TT	TT/TC	TC/TC	TT/CC	TC/CC	CC/CC
Total (n)	588	3	92	10	382	95	6
Percentage (%)	100	0.51	15.64	1.71	65.97	16.16	1.02
Average Age (years)	27	33	25.97	15.9	26.45	28.97	30.67
Female (n)	360	1	47	7	324	68	3
Female (n) %	61.22	0.27	13.05	1.94	90	18.89	0.83
Female Average Age	31	50	34.13	19.43	30.53	33.19	31
Male (n)	228	2	45	3	148	27	3
Male (n) %	38.78	0.88	19.74	1.31	64.9	11.84	1.32
Male Average Age	19	24.5	17.44	7.66	20.01	18.33	30.33

**Table 5:** The allele frequencies of *APOE* ε4, ε2 and intron 1 variations among different populations, globally retrieved from the NIAGADS Alzheimer’s Genomics Database, accessed on 1 September 2023 (https://www.niagads.org/genomics/app) [22].

Populations	ε4 Frequency	ε2 Frequency	Intron 1 Frequency
Global	0.151	0.075	0.626
African	0.268	0.103	0.881
Mixed American	0.104	0.048	0.578
East Asian	0.086	0.1	0.420
European	0.155	0.063	0.637
South Asian	0.087	0.044	0.509
European Finnish	0.327	0.074	0.775
Others	0.171	0.099	0.643
Turkish current study	0.0994	0.0918	0.70068

**Table 6:** The relative allele frequencies of non-common *APOE* SNPs across diverse populations and ethnic groups for comparison. Retrieved from dbSNP and ensemble genomic databases respectively (https://www.ncbi.nlm.nih.gov/snp/) (http://www.ensembl.org/Homo_sapiens/Info/Index) accessed on 8 February 2024.

Other *APOE* SNPs	Current Study	European	African	African American	East Asian	Latin American	South Asian	Others
rs373985746	0.0017	0.00008	0.0000	0.0000	0.018	0.000	0.00	0.0002
rs781722239	0.0017	0.00106	0.0005	0.0005	0.000	0.000	0.00	0.0011
rs762933906	0.0017	0.00030	0.0000	0.0000	0.000	0.000	0.00	0.0003
rs267606664	0.0017	0.002592	0.0003	0.0003	0.0000	0.001	0.000	0.00187
rs770562611	0.00085	0.00035	0.0000	0.0000	0.0000	0.000	0.00	0.0000
rs781722239	0.00085	0.000	0.000	0.000	0.000	0.000	0.000	0.000
rs267606664	0.00085	0.00016	0.0003	0.0003	0.000	0.000	0.00	0.0002
rs770562611	0.00085	0.000	0.000	0.000	0.000	0.000	0.000	0.000
rs1276509170	0.00085	0.000	0.000	0.000	0.000	0.000	0.00	0.000

**Table 7:** Cumulative Turkish data; the frequency of *APOE* gene alleles and genotypes within a healthy population across seven distinct studies.

Genotypes (rs429358/rs7214)	Total n (%)	1	2	3	4	5	6	7	Total (N)	%
	588 (100)	154	303	200	89	40	200	22	1008	
E2/E2 (TT/TT)	3 (0.51)	3	2	0	0	0	1	0	6	0.595
E2/E3 (TT/TC)	92 (15.64)	12	29	26	7	4	23	1	102	10.119
E2/E4 (TC/TC)	10 (1.71)	3	3	2	1	0	6	1	16	1.587
E3/E3 (TT/CC)	382 (65.97)	113	216	137	58	33	140	13	710	70.437
E3/E4 (TC/CC)	95 (16.16)	19	48	35	19	3	29	7	160	15.873
E4/E4 (CC/CC)	6 (1.02)	4	5	0	4	0	1	0	14	1.389
**Alleles**									**0**	**0**
E2	108 (9.18)	21	36	28	8	4	31	2	130	6.4488
E3	951 (80.86)	257	509	335	142	73	332	34	1682	83.433
E4	117 (9.94)	30	61	37	28	3	37	8	204	10.119
		154	303	200	89	40	200	22	1008	100

## Data Availability

Data available upon request.

## Abbreviations

The following abbreviations are found in this manuscript.

AD	Alzheimer’s Disease
*APOE*	Apolipoprotein E
SNPs	Single Nucleotide Polymorphisms
LOAD	Late-onset Alzheimer’s Disease
EOAD	Early-onset Alzheimer’s Disease
APP	Amyloid precursor protein
PSEN1	Presenilin-1
PSEN2	Presenilin-2
LDLR	Low-density lipoprotein receptor
LRP1	LDL Receptor Related Protein 1
BMD	Body Mass Index
CES	Clinical Exome Solution
BCL	Base Cell Binary
IGV	Integrative Genomic Viewer
WT	Wild Type
MUT	Mutant
TUSEB	Turkish Genome Project Data Sharing portal
CVD	Cardiovascular diseases
MTHFR	Methylenetetrahydrofolate reductase
ACE	Angiotensin-converting enzyme
Arg	Arginine
Cys	Cysteine
Avg	Average
